# The effects of orthognathic surgery on auditory function

**DOI:** 10.1186/s40902-021-00296-5

**Published:** 2021-03-25

**Authors:** Farhad Ghorbani, Hossein Danesteh, Afshin Khoramnia, Saeid Tavanafar

**Affiliations:** Department of Oral and Maxillofacial Surgery, School of Dentistry, Shiraz University of Medical Sciences, Shiraz, Iran

**Keywords:** Eustachian Tube Dysfunction Test, Eustachian tube, Orthognathic surgery, Pure tone audiometry, Osteotomy, Tympanometry

## Abstract

**Background:**

Orthognathic surgery is widely used in treating functional and skeletal problems. Any surgical procedure could cause side effects.

**Objectives:**

This study aimed to evaluate the potential changes in orthognathic surgery on the hearing function of patients.

**Materials and methods:**

Thirty-one orthognathic surgery candidates were recruited in this study. Patients underwent either single or double jaw surgery. Pure tone audiometry (PTA), tympanometry, and Eustachian Tube Dysfunction Test (ETFT) were performed postoperatively at 24 h, 6 weeks, and 6 months after surgery. Patients were tabulated based on the type of maxilla and mandibular surgical movements (vertical and horizontal).

**Results:**

PTA evaluation, based on horizontal or vertical movements, did not show significant differences, although vertical movements resulted in less change in hearing threshold. In other words, no significant changes occurred in patients’ hearing threshold after surgery. No significant difference was also observed between horizontal and vertical movements in the results of tympanometry. Negative changes were found in the results of ETFT in vertical movements, which returned to pre-surgery values in the final test.

**Conclusions:**

The risk of minor changes in hearing function is probable during the first week after orthognathic surgery, but these negative changes will either totally fade or remain negligible. Patients gave informed consent preoperatively, and reassurance postoperatively is prudent.

## Background

With increased life quality and life expectancy, the appreciation for facial esthetic has also improved. Although esthetic criteria are different among societies based on culture and social values, the dramatic increase in therapeutic surgical and non-surgical cosmetic procedures is evident. On the other hand, congenital anomalies, systemic diseases, syndromes, trauma, habits, etc. which cause developmental disorders can be an interfering factor for skeletal development of the face. These disorders can emerge as limited, asymmetric, or overgrowth of the jaws and facial skeleton.

Any surgical procedure could cause relative adverse outcomes. These could occur during or after the procedure and can be either minor or severe, transient or permanent, and idiopathic or iatrogenic. Orthognathic surgery due to its close relation with the nasopharynx, oropharynx, and temporomandibular joints can affect the neighboring anatomic structures such as the muscles of the soft palate, pharynx, Eustachian tube, masticatory muscles, and neurovascular structures [1–4]. The middle ear is posteriorly connected to the mastoid area and anteriorly to the nasopharynx through the Eustachian tube. The normal function of the Eustachian tube is necessary for normal middle ear function [5]. The muscles of the soft palate and pharynx are directly related to the hearing tube [6]; therefore, maxillary orthognathic surgeries either in the horizontal or vertical plane can change the anatomy of the soft palate and pharynx, affecting the Eustachian tube and consequently the middle ear’s function. Mandibular transpositions can affect the temporomandibular joint and also the position and tension of the muscles in the area. The masticatory muscles have anatomic proximities to the oropharynx and nasopharynx or even the Eustachian tube [7–9]. This could result in hearing changes after the maxilla or mandible (or both) movement during orthognathic surgery [4].

In the present study, we aimed to evaluate the decrease, increase, or stability of auditory function after orthognathic surgery and explore the causes and management of these problems. Also, the type of maxilla and mandible movement, either vertical or horizontal, and their effect on hearing function were investigated.

## Materials and methods

### Sample patients

The study protocol was approved by the ethical committee (Ethical Code ID: IR.SUMS.REC.1396.115). Thirty-one patients, including 14 men and 17 women, were recruited in this study. The inclusion criteria were the need for orthognathic treatment, minimum age of 17, no known ear pathology, no history of hearing problems, and willingness to participate in the study. The exclusion criteria of this study were patients with palatal clefts, hemifacial macrosomia, Pierre Robin sequence, Treacher-Collin syndrome, mental disorders; those with a history of hearing disorders or congenital hearing problems; patients with a history of receiving hearing treatments; and those who were not willing to participate in the study. The purposes and procedure of the study were explained to the participants, and informed consent was taken from all of the participants.

A treatment plan, using clinical and paraclinical information, was formulated, and the extent of the jaw repositioning with the surgery was agreed upon between the surgeon and orthodontist. All of the patients underwent hearing tests 24 h before, 6 weeks, and 6 months after the surgery. Pure tone audiometry (PTA), Eustachian Tube Dysfunction Test (ETFT), and tympanometry were used in the present study and performed in a certified audiometry center by an experienced audiometrist.

The surgical treatment plan for the participating patients in this study was either single or double jaw surgery with or without genioplasty. LeFort 1 osteotomy and bilateral sagittal split ramus osteotomy were chosen as the standard surgical procedure for mobilizing the maxilla and mandible, respectively. The mobile segments of the maxilla and mandible were rigidly fixed. Nasal intubation was applied for all of the patients. Before the surgery, all of the patients received 1 mg cefalotin sodium as an antibiotic and 8 mg of dexamethasone and continued for 3 days after the surgery. Patients underwent maxillo-mandibular rubber elastic traction starting 1 day after the surgery.

To evaluate the effect of jaw movements on hearing function, it was necessary to evaluate the maxillary and mandibular movements separately (advancement, set back, and upward and downward movements were separately evaluated for each jaw). Since the number of patients requiring pure unidirectional jaw movements was not enough, the patients were divided into the following groups for the assessment of the effect of jaw movements on hearing capacity:

A. Patients with only horizontal osteotomy repositioning (without vertical repositioning)
B. Patients with considerable vertical osteotomy repositioning (more than 2mm, with or without horizontal repositioning)

For the ease of discussion, absolute horizontal osteotomy repositioning is considered as horizontal, and patients who required repositioning in the vertical dimension (with or without horizontal repositioning) are considered as vertical.

### Hearing function tests

To perform pure tone audiometry (PTA), the patients were exposed to frequencies between 250 and 8000 Hz for each ear. A range of 0–20 dB in each frequency was considered as a normal hearing function. After the audiogram was obtained, the average intensity and the intensities obtained in 500 Hz, 1000 Hz, and 2000 Hz frequencies were reported as average PTA.

Tympanometry was obtained by recording a tympanogram for each ear; the compliance between 0.3 and 1.5 mm was considered as An diagram (normal tympanic membrane movement); the compliance between 0.1 and 0.3 mm was considered as As (little movement of the tympanic membrane); and the compliance less than 0.1 mm was considered as B diagram (stable tympanic membrane with no movements), which demonstrated middle ear effusion or a tear in the tympanic membrane. In diagram interpretation, if the pressure was less than −100 daPa (decapascals, 1.0 daPa = 10 Pa = 1.02 mm H_2_O), the diagram was considered as C, which demonstrated otitis media without effusion or in the early stages of the middle ear effusion. It is important to mention that in few cases with higher compliances than 1.5 mm, Ad was reported as a result of a thin tympanic membrane or spontaneous healing after the tear.

In Eustachian Tube Dysfunction Test (ETFT) for each ear, the measures according to the obtained diagram in normal, swallowing, and obstructed nose situation, as well as Valsalva maneuver, were recorded and interpreted into weak or normal.

### Statistical analysis

After performing the hearing tests in three periods for each patient, the results of the tests were sent to a statistician to be analyzed by RM ANOVA based on the changing process over time and intergroup comparison. Quantitative and qualitative variants were also analyzed among groups with the Mann-Whitney and chi-square tests. All the statistical calculations were done by the SPSS software, and a *p* value < 0.05 was considered statistically significant.

## Results

Table 1 shows the demographic information of participants. The mean age of the participants was 21.13±3.04 years (ranged between 17 and 30 years old). The patients’ distribution according to the type of deformity and jaw movement showed 15 patients (48.4%) presented only horizontal movements and 16 patients (51.6%) presented vertical movements with or without horizontal movements. The amount of movement in either plane ranges from 3 to 8mm. Larger movements were usually associated with craniofacial deformities and were excluded from this study.

**Table 1:** Descriptive statistics for demographic variables according to the type of surgery (one-jaw and two-jaw surgery)

		Type of surgery	
		One-jaw (*n*=6)	Two-jaw (*n*=25)
Age		25.51±3.54	20.08±3.00
Age range		(30–19)	(17–27)
Sex	Female	4 (67%)	17 (68%)
	Male	2 (33%)	8 (32%)

Age was described using mean ± standard deviation, and sex was described using frequency (%)

The following results were obtained from triple hearing tests.

### Pure tone audiometry

Table 2 shows the mean PTA test according to the type of movement (horizontal and vertical) for the right and left ears.

**Table 2:** Mean PTA during the time according to the type of deformity and movements in the right and left ear

		Pure tone audiometry (mean)		
		*T*_0_	*T*_1_	*T*_2_
Right ear	Horizontal	4.6±3.09	6.87±3.45	6.53±3.36
	Vertical	7.81±3.28	7.69±2.66	7.69±3.31
Left ear	Horizontal	5.54±3.59	6.31±4.23	6.21±3.70
	Vertical	7.49±2.99	8.52±3.16	6.81±3.11

The values in the table are mean±standard deviation

In the right ear, the comparison of vertical and horizontal movements and their effects on PTA showed that patients with vertical movements did not present any changes in PTA (*p*=0.233); although in the horizontal movement group, the mean PTA worsened over time, repeated measure ANOVA indicated that there was no statistically significant interaction effect between time and type of surgery (either vertical or horizontal movement, *p*=0.134). Regardless of the type of surgery, the mean PTA had no significant changes over time (*p*=0.198). Moreover, the mean PTA over time was not statistically different between the two types of surgery (*p*=0.079).

**Fig. 1:**
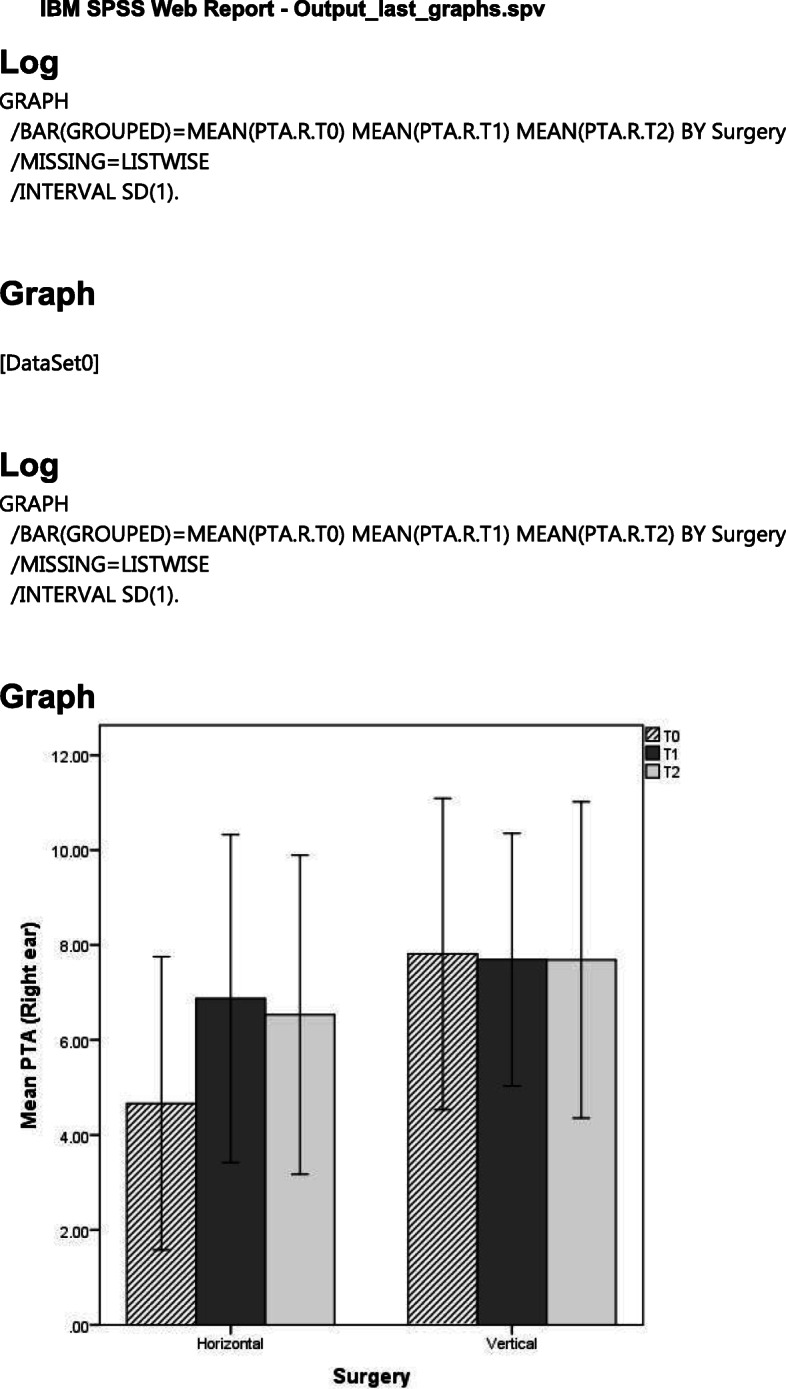
Mean PTA over time according to the type of movements (horizontal or vertical) in the right ear

**Fig. 2:**
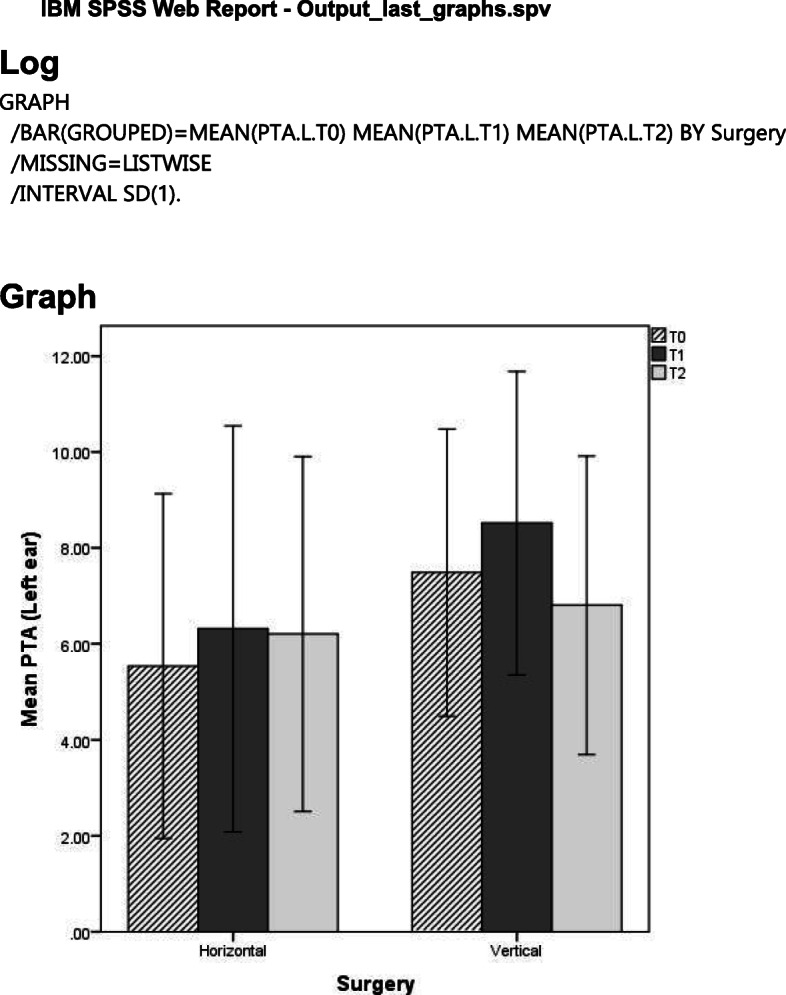
Mean PTA over time according to the type of movements (horizontal or vertical) in the left ear

Similar results were obtained for PTA of the left ear in terms of interaction effect (*p*=0.414), time of surgery (*p*=0.294), and type of surgery (0.118). Figures 1 and 2 illustrate the trend of the mean PTA overtime for the two types of movement in the left and right ears, respectively.

### Tympanometry

Generally, all of the patients (100%) presented type A tympanometry before the surgery in the right ear, and all of them (100%) again showed the same diagram after the surgery (specifically dividing, 77.5% presented An and 22.5% presented As, while after the surgery, these measures were 80.7% and 19.3%, respectively)

All the patients (100%) presented type A tympanometry before the operation in the left ear (specifically dividing, 84% presented An, 13% As, and 3% Ad), and these measures changed for 90.3% A tympanometry and 9.7% C tympanometry after the surgery. Table 3 shows the frequency of the type of tympanometry among patients.

**Table 3:** The distribution of tympanometry diagrams during the time in the right and left ears

Tympanometry	Right ear			Left ear		
	*T*_0_	*T*_1_	*T*_2_	*T*_0_	*T*_1_	*T*_2_
An	24	21	25	26	21	27
As	7	5	6	4	3	1
Ad	0	2	0	1	2	0
B	0	0	0	0	1	0
C	0	3	0	0	4	3
Total	31	31	31	31	31	31

The values in the table are frequency according to tympanometry diagrams

In a more detailed evaluation, no significant differences were observed between the right and left ears according to the type of jaw movements, in times *T*_0_, *T*_1_, and *T*_2_ (the *p* value for right and left ears was 0.394 and 0.226, respectively). However, it is essential to mention that three cases of type C tympanometry were observed in the left ear, which was related to the group with vertical movements.

### Eustachian Tube Dysfunction Test

The frequency of the type of ETFE (normal and poor) for both ears according to the type of movement is shown in Table 4. Horizontal and vertical movements of the right ear did not show significant differences in *T*_0_, *T*_1_, and *T*_2_ periods (*p*=0.10). Horizontal and vertical movements of the left ear did not reveal significant differences in *T*_0_, *T*_1_, and *T*_2_ periods (*p*=0.10), although in the *T*_1_ period, the left ear has shown more vertical (worsened) changes. Generally, the tympanic membrane in 93.5% of the right and 87% of the left ears were normal before the surgery. These measures for the right and left ears were 93.5% and 90.5%, respectively, 6 months after the surgery, which was nearly the same as pre-surgery measures (Table 4).

**Table 4:** The frequency of ETFE for the right and left ears according to the type of deformity and class of deformity during the time

Eustachian Tube Dysfunction Test							
		*T*_0_		*T*_1_		*T*_2_	
		*N*	*p*	*N*	*p*	*N*	*p*
Right ear	Horizontal	14	1	15	1	15	1
	Vertical	15	1	14	1	14	1
		29 (93.5%)	2 (6.5%)	29 (93.5%)	2 (6.5%)	29 (93.5%)	2 (6.5%)
Left ear	Horizontal	12	3	12	3	14	1
	Vertical	15	1	10	6	14	2
		27 (87%)	4 (13%)	22 (71%)	9 (29%)	28 (90.5%)	3 (9.5%)

## Discussion

The purpose of this study was to evaluate the potential impact of orthognathic surgery on hearing function. Several studies showed the relation of orthognathic surgery and alteration of hearing ability. Most of these studies considered the maxillary osteotomy more critical than the mandible. Changes in the anatomy of the soft palate and nasopharynx muscles as well as a change in direction and tension of para-tubular muscles, especially in advancement movements, are more emphasized when exploring maxillary surgeries [10]. A surgical procedure such as improper osteotomies of the pterygoid area, which is near the attachment of the hearing tube and operative muscle of the Eustachian tube can also interfere with normal auditory function. Factors such as trauma and the scar of the muscles around the hearing tube can reduce the hearing capacity [10].

Mandibular movements may probably affect the hearing capacity because of the proximity of the condyle and TMJ to the ear structures and common neurovascular systems of these areas [3, 11]. Furthermore, maxillary repositioning can change the hearing capacity through generating tension and edema in the masticatory muscle and, consequently, excess pressure on the adjacent structures [8, 9].

The impact of some general factors such as edema in the areas surrounding the hearing tube, which are of osteotomy surgery side effects or some procedures such as nasal intubations, on the function of the Eustachian tube is confirmed [10]. Some of the anesthetic agents in prolonged surgeries can cause the dysfunction of the Eustachian tube cilia and resultant hearing dysfuction [12].

The noises of the saw application and other rotary instruments during the osteotomy can negatively impact the hearing capacity [13]. The use of maxillo-mandibular fixation (MMF) and the immovability of the patients after the surgery can result in a reduction of the natural function of the nasopharynx area (like swallowing, speech, yawning) [4, 14].

One of the strengths of this study, in comparison with numerous similar articles, was the long follow-up period, so that the patients have nearly fully overcome the compulsory limitations of the surgery such as reduced physical activity and other functional limitations and have been living their routine lifestyle after 6 months. Also, both ears have been separately evaluated in this study, which was novel in comparison with other studies.

According to the PTA test, all of the patients, whether in 6 weeks or 6 months follow-up, presented a normal hearing threshold (>20 dB) although some fluctuations were observed. In the general evaluation (without considering the type of vertical or horizontal movement or a specific class of deformity), patients did not present tangible changes in their hearing threshold. In other words, orthognathic surgery did not have a long-term impact on the hearing threshold (Table 3). However, statistical evaluation was performed according to the type of vertical and horizontal movements; the patients presented better results in the right ear in the vertical movement group. The vertical movement in this study only included the maxillary impaction (patients did not undergo inferior maxillary reposition in this study); it seems that upward repositioning of the maxilla can act as a positive factor. Similar to the results of Wong et al.’s [9] study, in which they stated that maxillary elongation has a negative impact on hearing capacity, they mentioned maxillary advancement and elongation could cause hearing symptoms [9]. They also reported that among 74 ears that did not have any hearing loss or ear effusion, three ears consistently tested negative during the 6-month period of the study. They also reported less than 22.2% of ears had aural symptoms at 6 months post-operative period. The ears that experiencing fullness and otalgia returned to pre-operative status, while perceived hearing problem and tinnitus have significantly reduced. In their study, some of the patients reported persistent aural symptoms [9]. Algudkar et al. [10] reported a 22-year-old female with persistent bilateral middle ear effusion for more than 2 years after orthognathic surgery. The patient’s hearing loss was treated with grommet insertion while her rhinitis was treated with nasal steroids.

According to tympanometry, the A diagram was subdivided into Ad, An, and As for further evaluations. In our study, similar to Yaghmaei et al. [4] and Bayram et al. [1], all of the cases presented type A tympanometry before the surgery, but in 6 weeks of follow-up after the surgery, 13% of our cases presented type C tympanometry, while in this time period, Yaghmaei et al. [4] and Bayram et al. [1] presented 15% of type C tympanometry, similar to the results of the current study. In our study, after 6 months of follow-up, only 9.7% of the patients (in the left ear) still presented type C tympanometry, which is not statistically significant. In the study above, follow-ups were not longer than 1 to 2 months.

According to ETFT, this study showed similar results in pre-surgery tests and 6 months of follow-up tests based on Eustachian tube function. In other words, orthognathic surgery did not affect the function of the Eustachian tube. However, the function of the left Eustachian tube was reduced in the 6-week follow-up (29%). It could be the result of some factors such as edema, swallowing, and speech limitations since in 6 months of follow-up, the results of ETFT were similar to the pre-surgery measures. A 37% reduction in Eustachian tube function was observed among Yaghmaei et al.’s [4] patients, in the 6-week follow-up; one probable reason for this difference in the results of their study with ours may be the application of MMF procedure that was performed for Yaghmaei et al.’s [4] patients. It could be assumed that our patients resumed the natural functions such as swallowing, mastication, and yawning because MMF was not implemented for them. Therefore, the impact of MMF on the hearing threshold can be confirmed.

Generally, the results of this study showed that regardless of the fact that the type of jaw movements can have negative impacts on the hearing threshold of the patients, the nature of orthognathic surgery can cause slight changes in the hearing capacity, during the first weeks after the surgery, but these negative changes will either totally fade or remain in slight values that can be totally disregarded and do not need any medical interventions.

Previous follow-up of 132 orthognathic surgery patients over 10 years showed that 86.4% had no hearing symptoms, and even 7.6% stated that they could hear better than when did not have their surgery done [15]. Minor alteration in hearing sensation is predictable, and patients require information before surgery and reassurance after the surgery. This is consistent with previous studies [1, 4, 6, 9, 11].

Some of the present study limitations is more sample size of the patients should be investigated. Subjective reports of the symptoms could also obtain a more clear sight of the patients’ experience of hearing problems after orthognathic surgery.

## Conclusion

Orthognathic surgery can cause transient alterations in auditory function, which either totally fades away or remains negligible. Patients gave informed consent preoperatively, and reassurance postoperatively is prudent.

## Data Availability

The datasets used and/or analyzed during the current study are available from the corresponding author on reasonable request.

## Abbreviations

MMF: Maxillo-mandibular fixation
PTA: Pure tone audiometry
ETFT: Tympanometry and Eustachian Tube Dysfunction Test
